# Experimental Characterization of Polarized Light Backscattering in Fog Environments

**DOI:** 10.3390/s23218896

**Published:** 2023-11-01

**Authors:** Maria Ballesta-Garcia, Sara Peña-Gutiérrez, Pablo García-Gómez, Santiago Royo

**Affiliations:** 1 Centre de Desenvolupament de Sensors, Instrumentació i Sistemes, Universitat Politècnica de Catalunya (UPC-CD6), 10 Rambla Sant Nebridi, E08222 Terrassa, Spain; sara.pena.gutierrez@upc.edu (S.P.-G.); santiago.royo@upc.edu (S.R.); 2 Beamagine S.L., 16 C/Bellesguard, E08755 Castellbisbal, Spain; pablo.garcia@beamagine.com

**Keywords:** fog environments, polarization, imaging, polarimetric imaging, backscattering, turbid media, optical sensors, optical measurements

## Abstract

This paper focuses on the experimental characterization of the polarization behavior of light backscattered through fog. A polarimetric orthogonal state contrast imager and an active, purely polarized white illuminator system are used to evaluate both linear and circular polarization signals. The experiments are carried out in a macro-scale fog chamber under controlled artificial fog conditions. We explore the effect of backscattering in each imaging channel, and the persistence of both polarization signals as a function of meteorological visibility. We confirm the presence of the polarization memory effect with circularly polarized light, and, as a consequence, the maintenance of helicity in backscattering. Moreover, the circular cross-polarized channel is found to be the imaging channel less affected by fog backscattering. These results are useful and should be taken into account when considering active polarimetric imaging techniques for outdoor applications under foggy conditions.

## 1. Introduction

The detection and discrimination of objects inside fog is essential in fields like surveillance and navigation. In general, active optical systems are used for capturing the information needed for object identification [1]. A state-of-the-art challenge which arises in this domain is the loss of sensitivity in these systems under the presence of scattering media, such as fog. The key problem in this situation is the existence of a large amount of backscattered light from the media that acts as background light, which may blind or saturate the sensors. As a consequence, when imaging, contrast decreases and post-processing—including segmentation of objects—becomes a much harder task [2].

When light is sent through a turbid medium and subsequently collected, photons can be categorized into three main classes: ballistic, snake, and diffusion photons. This classification arises from the diverse mechanisms by which photons scatter during propagation. Each class conveys unique information about the medium and the scene, facilitating various imaging modalities [3].

Ballistic photons comprise the group of photons that are non-scattered and propagate strictly in a forward direction. They are responsible for regular reflection, and their capture is essential for achieving sharp images. Nevertheless, in media characterized by high scattering, as often encountered in turbid environments, the presence of these photons tends to be negligibly small. Snake photons include photons that experience a limited number of scattering events in forward or near-forward directions. Consequently, their paths within the medium are similar, but not identical, to ballistic photons. These photons are more abundant than their ballistic counterparts, enhancing their detectability. Finally, diffusion photons include those photons that have travelled through the medium over extended periods, following multi-step random paths, typically without interacting with the objects within the scene. They are not optimal for imaging purposes and they are often responsible for the large backscattering effect that can saturate the sensor. Under conditions of high scattering, diffusion photons predominate.

Therefore, it is paramount to capture an optimal number of ballistic and snake photons, which contain critical information pertaining to the scene obscured by fog. In pursuit of this objective, light-detection methodologies in turbid media prioritize the discerning of light that has traversed the media with minimal deviation and carries relevant information regarding the objects on a scene (ballistic and snake photons), as opposed to light that, due to extensive scattering, has largely been stripped of its original information and has only interacted with the medium (diffuse photons). By effectively distinguishing them, it becomes possible to enhance image contrast significantly [4,5,6]. Moreover, with the integration of advanced computational techniques, such as machine learning algorithms, further refinement and optimization in the detection of objects can be achieved [7,8]. This convergence of photon differentiation methods and cutting-edge computational tools heralds a new era of high-contrast, precision imaging.

The research domain encompassing these methodologies is extensive. Different approaches have been studied as a way to overcome this problem. Some authors have tested the efficiency of using polarization as a way to collect ballistic (and snake) photons and discriminate objects in foggy environments [9,10,11]. The use of polarization improves image contrast relaying of the polarimetric differences between reflected light from targets and backscattered light from media [12]. Therefore, an accurate characterization of the polarimetric characteristics of the medium of interest, in our case fog, is of special significance. In this paper, we seek to experimentally characterize the polarimetric properties of fog in the visible range for different visibilities by analyzing its backscattering response.

Fog is technically described as a medium containing spherical water particles smaller than 10 µm suspended in the air [13]. This phenomenon may lead to serious inconvenience for highly up-to-date outdoor applications which rely on imaging or computer vision, such as self-driving vehicles, including cars. Our objective is to give an insight into the backscattering response of the different polarization states for white light illumination, as would be emitted by conventional car headlights. These results will be useful as a compendium of information to consider when designing active systems for applications like polarimetric imaging, in tasks as diverse as terrestrial observation or transportation within foggy environments. Previous studies have been performed regarding forward scattering [14]; however, backscattering has not been studied in this way until now. The inherent characteristics and tendencies, along with the methodologies for their analysis, vary between transmission and reflection configurations. Moreover, in the context of forward scattering, measurements are long-ranged and the beam’s divergence is central to discussions surrounding light depolarization. Such considerations are not pertinent when examining the backscattering response. This underscores the significance of conducting a distinct and separate investigation of the latter.

Active polarimetric imaging systems illuminate a scene with a defined incident polarization state. There are different methods to recover the image using the detected information. One of them consists of separating the co-polarized and cross-polarized components of the detected light. The co-polarized component refers to the light with the same state of polarization as the initial illumination, whereas the cross-polarized component refers to the one with the orthogonal state. Image contrast is increased by combining the information of both components into a unique image [15,16]. In general, polarized light reflected from ordinary targets is mostly depolarized, with a smaller polarized component in the co-polarized state for linearly incident light and in the cross-polarized state for circularly incident light. Nevertheless, this dependence may vary on the specific material of the object [12,17].

For a turbid media, the polarization properties of backscattering can be quite different depending on the regime. According to scattering models, when the particle diameter α is slightly larger than the wavelength λ, i.e., α ≥ λ, Mie Theory can be used to describe the scattering. In this case, the incident light experiences a sequence of near-forward scattering events before it contributes to the backscattered light. That would be the case when working with visible light of λ ~ 500 nm in a fog of α ~ 1 μm. Under these circumstances, the linearly polarized light is depolarized rapidly, whereas the circularly polarized light is maintained for longer because the wave’s helicity is randomized less rapidly than its direction [18,19]. This phenomenon is known as the polarization memory effect.

The polarization memory effect was initially identified in a theoretical study focused on multiple scattering within optically dense media. This research documented the preservation of the polarization state, most notably with respect to circularly polarized light that was backscattered from turbid samples using the field correlation function [18]. Next, the phenomenon was studied experimentally by means of a time-resolved system [20]. This investigation contrasted two phantoms composed of scattering particles with disparate diameters. From their findings, it was deduced that in the phantom with larger particles, circularly polarized light had a “better” memory of its helicity. After that, researchers in the field of imaging through turbid media recognized the potential of this phenomenon, particularly in biomedical applications and microscopic imaging. They harnessed the preservation of circular polarization as a strategy to enhance image contrast [21,22].

Fog exhibits characteristics akin to the turbid media encountered in biomedical optics. Despite this, extending the conclusions found in other media to natural and real environments is not straightforward, as real fog may vary significantly from its theoretical behavior due to particle size distribution and temporal fluctuations. Efforts have been made to employ models and simulations to provide insights into this field [23]. However, to the best of our knowledge, nobody has experimentally characterized and accurately quantified backscattering in fog. This paper intends to give conclusive results on this topic. Upon completing this characterization, it becomes feasible to engineer imaging systems predicated on the polarimetric properties of fog. This is especially true if the memory effect is identified, offering an avenue to enhance image contrast.

## 2. Materials and Methods

**Figure 1:**
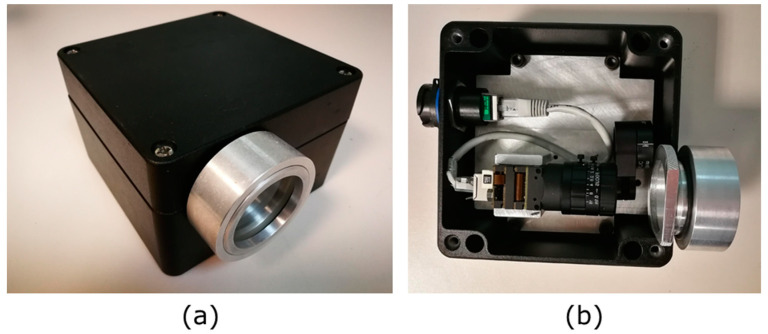
(**a**) Final encapsulation. (**b**) Camera encapsulated inside IP68 housing case protection.

We used a novel experimental system for measurement, composed of a polarization camera (Phoenix 5.0 MP Colour Polarization camera, LUCID Vision Labs, Richmond, BC, Canada), together with an objective of fixed focal length (EO #59-871) for analyzing polarization. The arrangement was placed inside an aluminum IP68 housing case for water protection, as shown in Figure 1.

**Figure 2:**
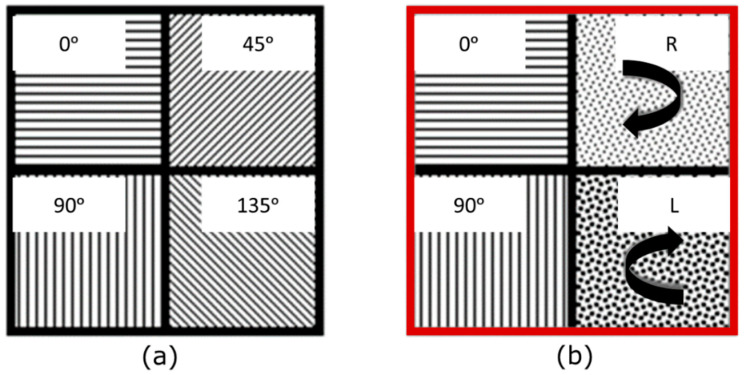
(**a**) Scheme of a macro pixel from the DOFP camera, comprising 4 micro-polarizers whose optical axes are oriented at 0°, 45°, 90°, and 135°. (**b**) Modification of the measuring states when placing the QWP (red border) in front of the DOFP camera. The channels corresponding to the angles 45° and 135° are modified by properly orienting the QWP to detect left and right-handed circular polarization, respectively. Linear channels 0° and 90° remain unchanged.

The camera, which is based on a CMOS sensor (IMX250MYR, Sony, Tokyo, Japan), enables polarimetric imaging through a division of focal plane arrangement (DOFP). The sensor of the camera consists of an array of linear micro-polarizers, oriented at four different angles of polarization from the basis of the sensor (horizontal or 0° (HL), vertical or 90° (VL), 45°, and 135°). For the experiments, the imaging unit was modified with the introduction of an achromatic quarter waveplate (QWP) (AQWP10M-580, Thorlabs, Newton, NJ, USA) placed in front of the objective and aligned with the optical axis of the micro-polarizer at 0°. The calibration comprised two steps. A first radiometric calibration addressed the non-uniformity in the transmission of the micro-polarizers. Subsequently, the polarimetric calibration was undertaken to guarantee the accurate alignment of the QWP. When calibrated, the unit allowed the measurement of HL and VL polarization states, and also, simultaneously, the circular polarization states: right-handed (RC) and left-handed (LC) [14]. Obtaining HL, VL, RC, and LC simultaneously allows very stable measurements as long as the imaging system does not need any manual manipulation once it is set. Schematics of the changes in the sensor are made explicit in Figure 2. With these modifications, the DOFP camera became a snapshot orthogonal state contrast imager, able to compare the degree of linear (DOLP) and circular (DOCP) polarization at each shot. The described setup was used as the polarization state analyzer (PSA) in our setup.

**Figure 3:**
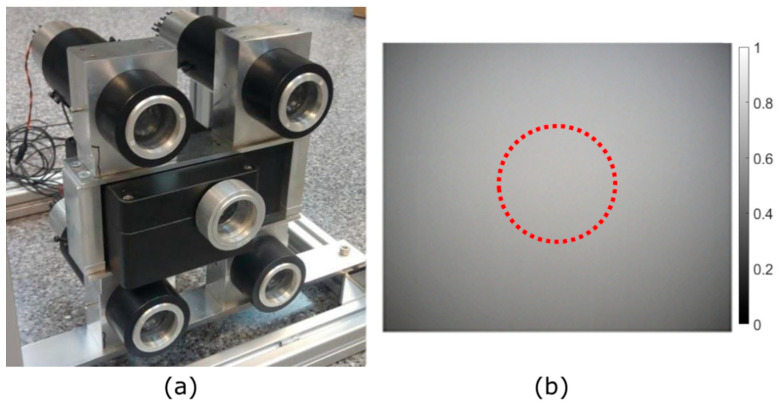
(**a**) Array of 4 active illuminators mechanically assembled around the camera housing. The final unit used in the tests. (**b**) The backscattering signal of the illuminator under dense fog conditions is produced by the illumination distribution received in the 4 polarization channels of the DOFP camera. The homogeneous light distribution in the ROI (red circle) is produced by the 2 × 2 configuration illuminator array.

A polarized light source (LS) was built, with an array of 4 active illuminators mechanically assembled around the camera housing. The final arrangement is shown in Figure 3a. The number of illuminators and their distribution was designed to produce a homogeneous illumination. In Figure 3b, it is shown how the combination of the four illuminators allowed homogeneous light distribution in a central Region of Interest (ROI) of 200 pixels of radius, centered on the middle of the field of view (FOV) of the camera.

Each illuminator consisted of a white light source with combining wavelengths from 400 to 750 nm using LED arrays (LUXEON Rebel ES LXML-PWC2, Lumileds, Amsterdam, The Netherlands), followed by a polarizer which worked as the polarization state generator (PSG). The polarization of the emitted light was manually interchangeable between HL and LC by using the corresponding polarizer (either circular or linear).

Since the illumination state can be either linear or circular, we define the channels of the camera as co-polarized (CO) for the channel in the PSA matching the active illumination, and cross-polarized (CROSS) for the orthogonal state regarding the polarization of the active illumination. In this way, when illuminating with a HL state, the CO channel is detecting HL polarization at the PSA, whereas the CROSS channel detects VL. Analogously, when the illumination is set to LC, the CO channel is associated with LC polarization, and the CROSS channel to RC.

**Figure 4:**
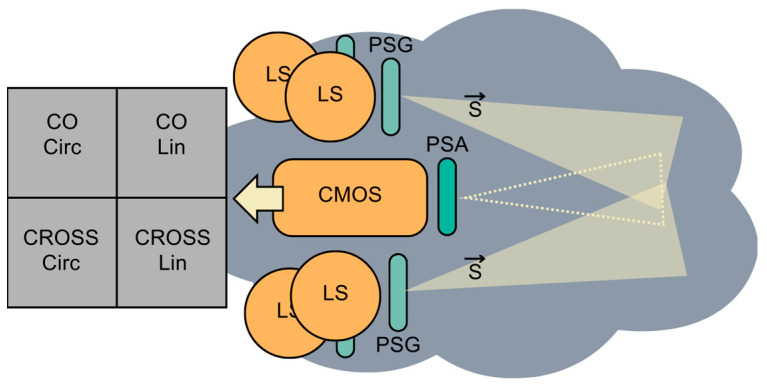
Scheme of the experiment where active polarized illumination is backscattered by fog and reaches the DOFP camera. LS, light source; PSG, polarization state generator; PSA, polarization state analyzer; $\vec{S}$, input polarization illumination.

Tests consisted of illuminating the fog chamber using reflection geometry (with detection performed from the position of the illuminator) with polarized light, either linear or circular. Backscattering properties were analyzed using the images acquired by the modified DOFP camera, which was in the same geometrical plane as the illumination (see Figure 3). The scheme of the setup used in the experiments is presented in Figure 4.

Experimental work was performed in a large-scale (30 m) fog chamber at CEREMA’s facilities, located in Clermont-Ferrand (France) [24]. Inside the chamber, homogeneous fog can be produced, with controlled and constant meteorological visibility. Visibility stands for the transparency of the atmosphere and is represented by the meteorological optical range (MOR). The MOR denotes the length of the path at which the luminous flux from a collimated beam emitted by an incandescent lamp, with a color temperature of 2700 K, is reduced to 5% of its initial value. This luminous flux is assessed using the photometric luminosity function established by the International Commission on Illumination [25]. In this case, the visibility is monitored using a separate transmissometer and measured in real-time inside the fog chamber, as explained in [26]. Visibility may be varied at will in different types of cycles. In this case study, cycles of fog with visibilities ranging from 15 m to 100 m were measured. The size of the water particle creating the fog may also be selected, ranging from small (~1 µm) to large (>10 µm) diameters. For our experiment, the mean particle diameter was 1 µm with a distribution very similar to the statistics of natural radiation fog [12]. All the tests were performed in night-time conditions, to avoid undesired light interactions.

The investigation of scattering caused by alternative particulates would also be of significance, yet it remains beyond the purview of this specific study. In our research, we did not account for other suspended entities such as dust (encompassing larger and irregularly shaped particles) or atmospheric gases (representing finer particles). Noticeably, the results of this study will change, as other kinds of particles may lead to other kinds of scattering. For example, small particles lead to Rayleigh scattering, while big particles account for non-selective scattering. Nevertheless, as experiments were performed using a controlled condition, in this case, fog conditions, the main scatterers were water droplets.

Two cases were considered: circular polarization (CP) and linear polarization (LP). The luminous flux emitted was adjusted to be the same in both polarizations, and the camera was set to 1 s of exposure time and no gain. During the experiments, the fog chamber was completely emptied of objects to avoid back-reflections. Therefore, the FOV of the camera did not contain any object. In each testing cycle, the chamber was filled with different densities of fog and was illuminated with purely polarized light (with a degree of polarization DOP > 98%), which is represented by a Stokes vector $\vec{S}$ in Figure 4. Since the polarization can be adjusted to HL and LC with the same characteristics, experiments that aimed to compare the backscattering of fog under the same illumination conditions, but with different polarization states at different visibilities were straightforward to perform.

## 3. Results and Discussion

**Figure 5:**
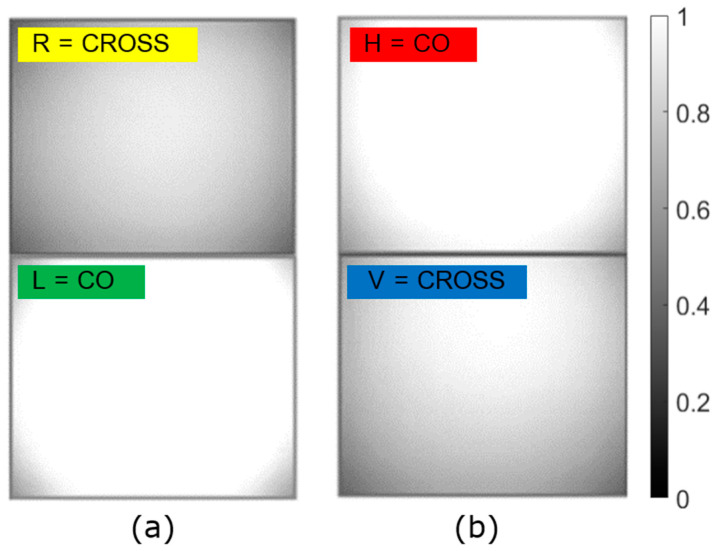
Irradiance detected in CO and CROSS channels for very dense fog (visibility < 15 m) when the illumination and detection pairs are (**a**) circularly polarized and (**b**) linearly polarized. The saturation in the CO channels remarks the prevalence of the input polarization state.

When imaging an empty medium with active illumination, lower visibility (or high density of scattering media) brings on a stronger detected irradiance due to directly backscattered light. In Figure 5, we show images of the CO and CROSS channels of the modified DOFP camera for (a) CP and (b) LP illumination and detection pairs, in extremely short visibility conditions (below 15 m). In both cases, the most saturated image corresponds to the CO channel. This implies that backscattered light in dense fog essentially keeps the same polarization state as the initial illumination. It can be concluded that when imaging under this condition, for both polarizations, the CO component of backscattered light would have the main responsibility of hampering the perception of the camera. As a consequence, CROSS channels seem to be good candidates to filter out most of this first backscattering effect.

**Figure 6:**
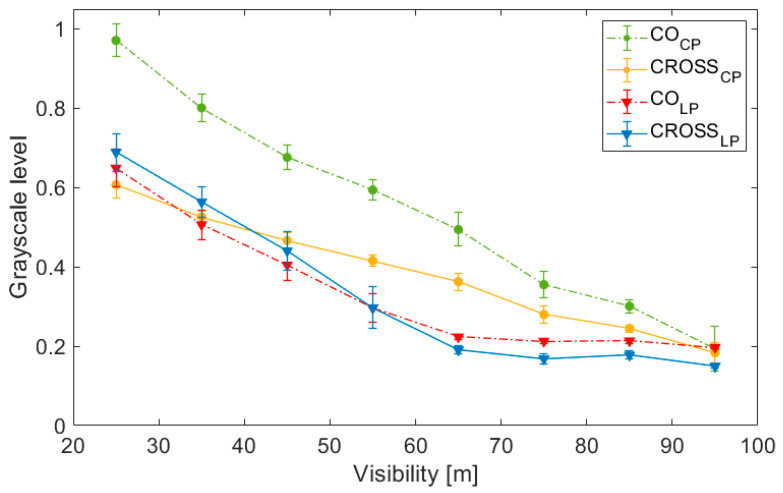
Mean irradiance values—averaged over time in increments of 10 m of visibility—of the light backscattered by fog for each polarization channel (CO and CROSS), for both circular (CP) and linear polarization (LP), as a function of the visibility.

Figure 6 shows the mean detected irradiance, averaged over time in increments of 10 m of visibility, of the central ROI as a function of visibility for CO and CROSS channels, for both circular and linear illumination and detection pairs. Each image has a size of 1224 × 1024 pixels, and the ROI is a circular region of 200 pixels of radius centered in the middle of the image, with an irradiance standard deviation of 0.0625. The ROI is indicated in Figure 3b using a red dotted circle. Also note that when the detected irradiance in the grey-scale is below 0.25, a discussion is no longer valid, because the amount of backscattered light received from the medium is not considered significant anymore. In other words, at that level, the fog density is not enough to produce sufficient scattering to cause significant backscattering interference in the imaged scene.

When irradiance values of CO and CROSS channels coincide, polarization is completely randomized since we are receiving the same amount of energy in both channels. Hence, light is depolarized. It can be appreciated that for the circular case, the mean grayscale levels of both channels are not evened until visibility reaches large values (low fog density and small scattering). Thus, for CP, a part of the backscattered light maintains the polarization even when visibility reaches 75 m. Instead, for LP, CO and CROSS signals are comparable in irradiance at 25 m visibility, because their difference is below 0.1 grey-level. Thus, at this level of visibility, linearly polarized light is already depolarized. When scattering media is very dense, fewer scattering events are needed to contribute to the backscattering effect. As the medium becomes less dense, light contributing to backscattering likely travels further before coming back. Relating visibility to the potentially travelled distance of light, we conclude that circular polarization is expected to maintain polarization at least three times further than linear polarization, with polarization being rapidly lost during the latter.

This observation is in accordance with the scattering models and proves that fog behaves according to Mie Theory for α ≥ λ, even for a variable range of particle sizes and wavelength and with the existence of temporal fluctuations [18,19]. Thus, it is confirmed that linear states are depolarized faster than circular ones, which are preserved due to the slower randomization of helicity, experimentally confirming the idea of a larger fog’s memory effect for circular polarization.

We can also observe in Figure 6 that the mean grey-level of the CROSS circular channel (yellow line) is lower than the CROSS linear channel (blue line) at low visibilities. The CROSS linear channel presents higher irradiance values because LP is depolarized faster. This effect could bring on an advantage in the CROSS circular polarization state, as long as it appears to be receiving less backscattered energy than its linear counterpart for a wider range of visibilities (up to 40 m of visibility), and not only for very dense fog, as was discussed in Figure 5. Hence, between the two CROSS channels, results confirm that the CROSS circular channel is always the channel less influenced by fog’s backscattering.

These results have major implications for the use of polarimetric imaging. For imaging, whenever we know that the elements of the scene will behave like ordinary targets (metals, ground, living beings, etc.)—which are either depolarizing for mostly dielectric surfaces, or reflecting co-polarized state for LP incident light and cross-polarized state for CP incident light, in the case of metallic surfaces [12,17]—the CROSS circular channel is going to show the best performance, with the maximum image contrast. So, in the CROSS circular case, while targets will mainly return non-polarized or circularly cross-polarized light, and thus, their signal will be detected, the media response will be filtered because the signal will mainly be in the circularly co-polarized component. In the LP case, using the CROSS linear channel to filter out fog backscattering also involves filtering out part of the polarized component of reflected light coming back from objects.

**Figure 7:**
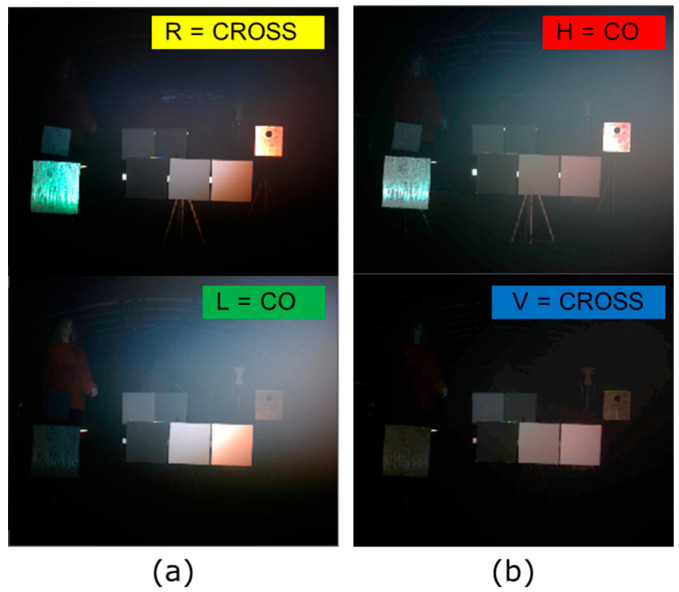
Images of the fog chamber with the presence of objects and dynamic fog for (**a**) CP and (**b**) LP illumination and detection pairs.

As an example, in Figure 7, we show the images of the CO and CROSS channels of the modified DOFP camera for (a) CP and (b) LP illumination and detection pairs when different objects were introduced inside the fog chamber, and our set-up was used to image them. The scene includes two sets of three calibrated plates (with 0%, 50%, and 100% reflectivity) in the center, three metallic plates on the sides, and two mannequins wearing different fabrics, also on the sides. In this case, fog and visibility were not controlled, because we wanted to reproduce a real environment and the dynamics of fog created irregularities in the media.

The main characteristics mentioned previously can be seen. CROSS CP (R) filters out most of the fog backscattering effect (image whitening) and at the same time returns a lot of signal from objects, especially metallic ones. CROSS LP (V) is the darkest image because both backscattering light from fog and reflected polarized light from objects are being filtered out. CO CR (L) collects backscattered light from fog and hides some of the objects, especially metal objects. Finally, CO LP (H) receives both backscattered light and light reflected from objects.

**Figure 8:**
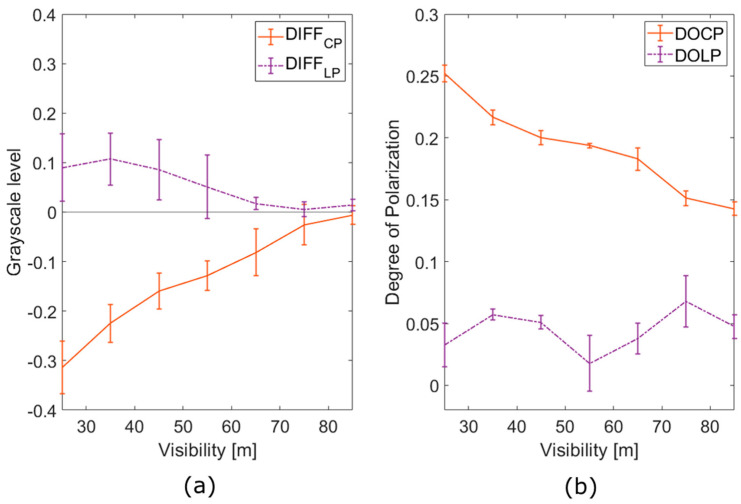
(**a**) The difference between the mean irradiance values of polarization channels (CROSS–CO) of the backscattered light by fog for each polarization state (C for circular and L for linear), as a function of the visibility. (**b**) The DOCP for the case of CP, and the DOLP for the case of LP, as a function of the visibility.

In Figure 8a, the mean difference (DIFF) between the CROSS and CO channels’ (CROSS–CO) values for each pixel inside ROI, averaged over time in increments of 10 m of visibility for the empty fog chamber, is depicted for both polarizations (CP and LP). We have used DIFF as a figure of merit to show the contrast between channels and polarization persistence. The larger the distance to zero, the greater the contrast between channels. Negative values of DIFF show the superiority of the use of the CROSS channel over the CO channel. In Figure 8b, the degree of polarization for both circular polarization (DOCP) and linear polarization (DOLP) is shown.

Based on the data presented in Figure 8, it is evident that CP demonstrates superior polarimetric performance in comparison to LP, as expected. In the case of CP, there exists a clear difference between the CO and CROSS channels, shown by larger DIFF. The signal consistently retains a polarized portion of light across the entire spectrum of examined visibilities, as indicated by the more pronounced DOCP. Instead, for LP, the DIFF parameter is almost negligible across all visibility levels, and similarly, DOLP remains minimal.

A significant observation from Figure 8a highlights the manner in which both polarizations respond to increased visibility, which corresponds to a decreasing density of the scattering medium. Both DIFF values exhibit a linear trend as a function of fog density, suggesting a linear depolarization pattern. The slopes derived from this trend can be used to characterize polarimetric behavior across various optical densities (OD) for a given medium. Notably, the absolute value of the CP slope surpasses that of the LP, providing an additional benefit for the use of CP by demonstrating the showing of a larger dynamic range.

It is also interesting to study the depolarization behavior of both signals. From Figure 8b, one can notice that there is a substantial depolarization of the detected light, even under conditions of high visibility. At 25 m of visibility, only 25% of CP light remains polarized, a significant reduction from the initial 98% of DOCP. However, this retained 0.25 DOCP is still detectable and can be leveraged to enhance contrast in the imaging process. In contrast, LP experiences complete depolarization across the entire range of visibilities studied. This depolarization accounts for the DIFF parameter remaining below 0.1, resulting in a signal characterized by an almost imperceptible degree of polarization. Consequently, although contrast differences between channels may offer some additional information, no substantial insights can be derived from the DOLP.

These findings are consistent with well-established scattering models. The randomization of helicity in CP occurs at a slower rate, requiring a greater number of interactions in order to be fully eliminated. Conversely, in the case of LP, the randomization process is contingent on a different mechanism associated with the randomization of direction and, consequently, the plane of linear polarization. This latter process necessitates only a few interactions to induce complete alteration.

Finally, the difference in detectivity for CP across all values of visibility—shown in Figure 8—highlights the opportunity to calculate parameters other than CO, CROSS or irradiance when looking for contrast improvement, such as parameters that incorporate the difference between both channels, like the use of DIFF or DOCP in a single image. These images could be used as a tool to emphasize the different polarimetric responses of the elements of a scene, and even open the possibility to develop dehazing algorithms based on them. However, in the case of LP, the use of the combination of CROSS and CO channels would add a less significant advantage, since the dynamic range will be highly reduced. Thus, we conclude that the analyzed data shows quantitative experimental evidence of the superiority of the circular polarization state for active imaging proposes.

## 4. Conclusions

The challenge of object detection and discrimination within foggy environments has been a persistent concern across several areas, particularly in the fields of navigation and surveillance. The primary obstacle is the backscattering effect of light in the presence of fog, which can severely impair the image quality. This research delved deep into the polarimetric properties of fog and the potential advantages of polarimetric imaging under different polarization states, particularly using CP.

Our findings indicate that in extremely dense fog conditions (visibility below 15 m), the co-polarized component of backscattered light is primarily responsible for obstructing camera perception, for both LP and CP. This suggests that CROSS channels offer a well-suited solution to filtering out most of the immediate backscattering effects.

When analyzing the irradiance values of the CO and CROSS channels for both polarizations, a significant observation emerged: circularly polarized light maintains its polarization state for up to three times longer than linearly polarized light under foggy conditions. Such behavior provides significant advantages for imaging, since the fog’s backscattering influence can be minimized. Additionally, CP also retains a higher detectable degree of polarization (DOCP) than LP does, even in decreased visibilities. This difference in behavior has implications not just for imaging quality, but also for the potential development of advanced imaging techniques and dehazing algorithms.

Such outcomes are congruent with scattering models in the α ≥ λ domain, revealing that fog behaves predictably even when accounting for the slight variety of particle sizes, wavelengths, and the presence of temporal fluctuations. Specifically, circularly polarized light preserves its polarization state for longer due to the slower randomization of its helicity, as opposed to linearly polarized light, which depolarizes rapidly. This observed memory effect of circular polarization, which has previously proven beneficial in other scattering media, could potentially be harnessed in applications dealing with fog.

With that, the advantages of using CP CROSS for detection are affirmed: they effectively filtered out the fog’s backscattering while retaining a substantial amount of signal from the objects in the scene. This contrasts sharply with LP, where both backscattered light from fog and reflected polarized light from objects were significantly diminished when using CROSS detection.

While the results are promising, the adoption of polarimetric imaging, particularly that employing CP, in real-world applications could be a game-changer. Consider self-driving vehicles navigating through dense fog, or surveillance systems operating in challenging weather conditions; the advantages of clearer imaging and enhanced object discrimination become clear. By filtering out the majority of fog interference while still capturing relevant data from the scene, the safety and efficiency of these applications could see significant improvements.

In closing, we have proved experimentally and quantitatively that white light behaves according to the scattering models when interacting with fog by using an active polarimetric imaging system and different kinds of analysis. LP is seen to be rapidly depolarized, whereas circular polarization is maintained for longer ranges, up to three times further than linear polarization. Thus, the wave’s direction is randomized faster than its helicity. We have also discussed the potential of taking advantage of the polarization’s memory effect for developing new imaging systems. Circular polarization may improve contrast under fog or other scattering conditions since (i) contrast between mean irradiance values of orthogonal channels is remarkable for a longer range of visibilities; (ii) the backscattering effect can be mitigated using the CROSS channel because it can filter out most of the backscattering signal, and thus sensor blinding due to saturation may be avoided. As industries continue to rely heavily on high-quality imaging, especially under challenging environmental conditions, insights from this study could pave the way for advancements in imaging technology, ensuring clearer, safer, and more efficient operations across a multitude of applications.

## Data Availability

Data underlying the results presented in this paper are not publicly available at this time as they were partly obtained through a privately funded project, but may be obtained from the authors upon reasonable request.
